# Clinical characteristics of Gram-negative and Gram-positive bacterial infection in acute cholangitis: a retrospective observational study

**DOI:** 10.1186/s12879-021-06964-1

**Published:** 2022-03-20

**Authors:** Shijing Tian, Kaili Li, Hong Tang, Yan Peng, Liang Xia, Xi Wang, Xiaoying Chen, Fachun Zhou

**Affiliations:** Department of Critical Care Medicine, The First Affiliated Hospital of Chongqing Medical University, 1 Youyi Rd, Yuzhong Qu, Chongqing, 400016 People’s Republic of China

**Keywords:** Acute cholangitis, Pathogen, Clinical features, Organ dysfunction, Prognosis

## Abstract

**Background:**

To investigate the difference in the severity of illness, organ dysfunction, and prognosis of acute cholangitis due to different pathogenic bacterial infection types.

**Methods:**

A retrospective observational study was performed. Patients who met the selection criteria according to blood culture and bile culture results of different pathogenic bacterial were divided into groups. The severity of illness, organ dysfunction, and prognosis of the groups were analyzed and compared comprehensively.

**Results:**

A total of 424 patients were included, and no bacterial growth developed in 111 patients (26.2%). Among the 313 patients (73.8%) with bacterial growth, 155 patients had only Gram-negative bacteria cultured (49.5%), 48 patients had only Gram-positive bacteria cultured (15.3%), and 110 patients had both Gram-negative and Gram-positive bacteria cultured (35.1%). The proportion of Grade III patients and the APACHE II and SOFA scores of the mixed Gram-negative and positive group were the highest (p < 0.05); the intensive care unit admission day and hospital stay were longer, and the mortality rate were also higher 20/110 (18.2%) than the other two groups. Regression analysis showed that bacterial growth was an independent risk factor for organ dysfunction. The risks of an increased septic shock, neurological dysfunction, hepatic dysfunction, hematological dysfunction, and respiratory dysfunction in the mixed Gram-negative and positive group were higher than the Gram-negative group (P < 0.05). The Cox proportional hazards regression prompt showed that different culture results were independent risk factors for death. The mixed Gram-negative and positive group had increased hazard ratios and 95% CI of 7.30 (95% CI 1.55 to 34.38) compared with the Gram-negative group. There was no difference between the Gram-negative group and the Gram-positive group in the severity of illness, organ dysfunction, intensive care unit admission day, hospital stay, mortality rate, and risk of death (P > 0.05).

**Conclusions:**

In acute cholangitis, mixed infection with Gram-negative and Gram-positive bacteria was more severe and was associated with a higher risk of death. There were no apparent differences between Gram-negative and Gram-positive bacterial infections.

**Supplementary Information:**

The online version contains supplementary material available at 10.1186/s12879-021-06964-1.

## Background

Acute cholangitis is an inflammation of the bile duct caused by biliary tract bacterial infection due to biliary tract obstruction. It is commonly related to biliary stones and biliary tract tumors; it can cause septic shock and multiple organ dysfunction in severe cases. Acute cholangitis has a high mortality rate up to 10% even after aggressive treatment [1]. Timely biliary drainage and the use of appropriate antibiotics are the keys to improving the prognosis [2, 3].

The pathogens of acute cholangitis ascend from the intestinal tract, mainly Gram-negative bacilli and Gram-positive cocci. Patients could be infected by either a single pathogen or a mixture of Gram-negative and Gram-positive bacteria. Both Gram-negative and Gram-positive bacteria can cause serious infections, resulting in sepsis or septic shock [4, 5]. Currently, it is difficult for clinicians to determine the type of pathogens in acute cholangitis patients. The 2013 and 2018 Tokyo guidelines state that if a patient develops severe cholangitis, the choice of empiric antibiotics should be anti-Gram-negative bacteria while covering Gram-positive bacteria [6, 7]. The guidelines emphasized that severe cholangitis could be due to a mixed infection of Gram-negative and Gram-positive, but there is no evidence to support it. After reviewing the literature, only a few reports compare the clinical features of Gram-negative and Gram-positive bacteria in acute cholangitis. There are no reports on the comparison of mixed infections of Gram-negative and Gram-positive bacteria vs. monomicrobial bacterial infections in acute cholangitis.

This study used retrospective data collection and analysis for two purposes: (1) to compare between Gram-negative and Gram-positive infections in acute cholangitis in terms of severity and organ dysfunction to provide evidence support for guidelines and to suggest proper empirical antibiotics; (2) to provide a prognostic evaluation of different types of bacterial infections in acute cholangitis and early prognostic judgment.

## Methods

### Study setting

In this retrospective observational study, all patients diagnosed with acute cholangitis admitted to the First Affiliated Hospital of Chongqing Medical University from July 2013 to July 2020 were investigated. The First Affiliated Hospital of Chongqing Medical University (CHMU) is a Grade III, Grade A general hospital in China. The Institutional Review Board of the First Affiliated Hospital of Chongqing Medical University approved this study.

### Patient selection

Patients with community-acquired infection of acute cholangitis who met the diagnostic criteria of Tokyo Guidelines from July 2013 to July 2020 were selected [8]. All enrolled patients received standardized treatment in accordance with the Tokyo Guidelines [9], including the treatment with antimicrobials and anti-shock immediately, combined with ERCP (Endoscopic Retrograde Cholangiopancreatography) or surgical drainage to remove the biliary obstruction as soon as possible. Blood cultures or bile cultures were taken from all patients, and the blood samples were drawn before antibiotics administration. Bile was collected from patients for bile culture during surgery. Some patients were excluded due to: (a) being under the age of 18 years; (b) had no ERCP or surgical drainage performed; (c) no bile culture and blood culture taken; (d) fungal infection; (e) treatment was given in other hospitals before admission; (f) organ failure before onset; (g) abandoned treatment or transfer to other hospitals.

According to the pathological results of blood culture and bile culture, enrolled cases were divided into a No-growth group (no bacteria were cultured from bile and blood) and a bacterial growth group (bacteria were cultured from bile or blood). The bacterial growth group was divided into three subgroups: a Gram-negative group (only Gram-negative bacteria were cultured from bile and blood), a Gram-positive group (only Gram-positive bacteria were cultured from bile and blood), and a mixed Gram negative and positive group (Gram-negative and Gram-positive bacteria were cultured from bile or blood, including bile and blood culture of one is Gram-negative bacteria and the other is Gram-positive bacteria).

### Data collection

The following clinical data were collected from each patient (a) age and gender; (b) co-morbidities/past medical history (cardiovascular disease, chronic pulmonary disease, history of malignancies, diabetes mellitus, chronic liver disease, chronic renal insufficiency, neurologic disorder, connective tissue disease); (c) history of any biliary procedures (including cholecystectomy, biliary stent placement, and biliary anastomosis); (d) the etiology of cholangitis (stones, tumor, or biliary stricture); (e) recurrence history; (f) clinical symptoms (fever, abdominal pain, and jaundice); (g) admission of appropriate initial antimicrobial therapy or not, which was defined as immediate admission of antimicrobials covering the causative microorganisms of blood or bile cultures; (h) biliary drainage procedure (ERCP or surgical operation); (i) the degree of illness (according to the Tokyo Guidelines cholangitis condition classification standard [8]); (j) blood or bile culture results; (k) organ function impairment; (l) intensive care unit admission time; (m) the length of hospital stay; (n) the prognosis (death or recovery). APACHE II (Acute Physiology And Chronic Health Evaluation II) and SOFA (Sequential Organ Failure Assessment) scores were performed on patient admission to evaluate the degree of illness and organ damage.

### Definitions used

Organ function impairment in this study was defined according to the Tokyo Guidelines organ damage standards [8], which is neurological dysfunction with disturbance of consciousness, respiratory dysfunction: PaO2/FiO2 < 300, renal dysfunction with oliguria and serum creatinine > 2.0 mg/ dL, hepatic dysfunction with PT-INR > 1.5, and hematological dysfunction with platelet count < 100,000/mm3.

SEPSIS-3 criteria were used to define septic shock [10] as vasoactive drugs were still needed under adequate fluid resuscitation to maintain mean arterial pressure greater than or equal to 65 mmHg.

### Statistical analysis

SPSS 26.0 software (IBM, Armonk, NY, USA) was used for data processing and GraphPad drawing. The measured data were tested for normality by the explorative method. When the normal distribution was presented as mean ± standard deviation (x ± s), the non-parametric Kruskal–Wallis test was used to compare the groups when the normal distribution was not presented as median (quant). Enumeration data were expressed as frequency (percentage) and compared the groups using χ2 or Fisher’s exact test. The binary logistic regression model was used to analyze the risk of different pathogenic culture results on organ dysfunction. A Cox proportional-hazards regression model was used to assess the influence of different cultures on the risk of death. Cumulative probability curves were constructed for subjects with different culture results. P < 0.05 was considered statistically significant.

## Results

### Patient characteristics

**Fig. 1:**
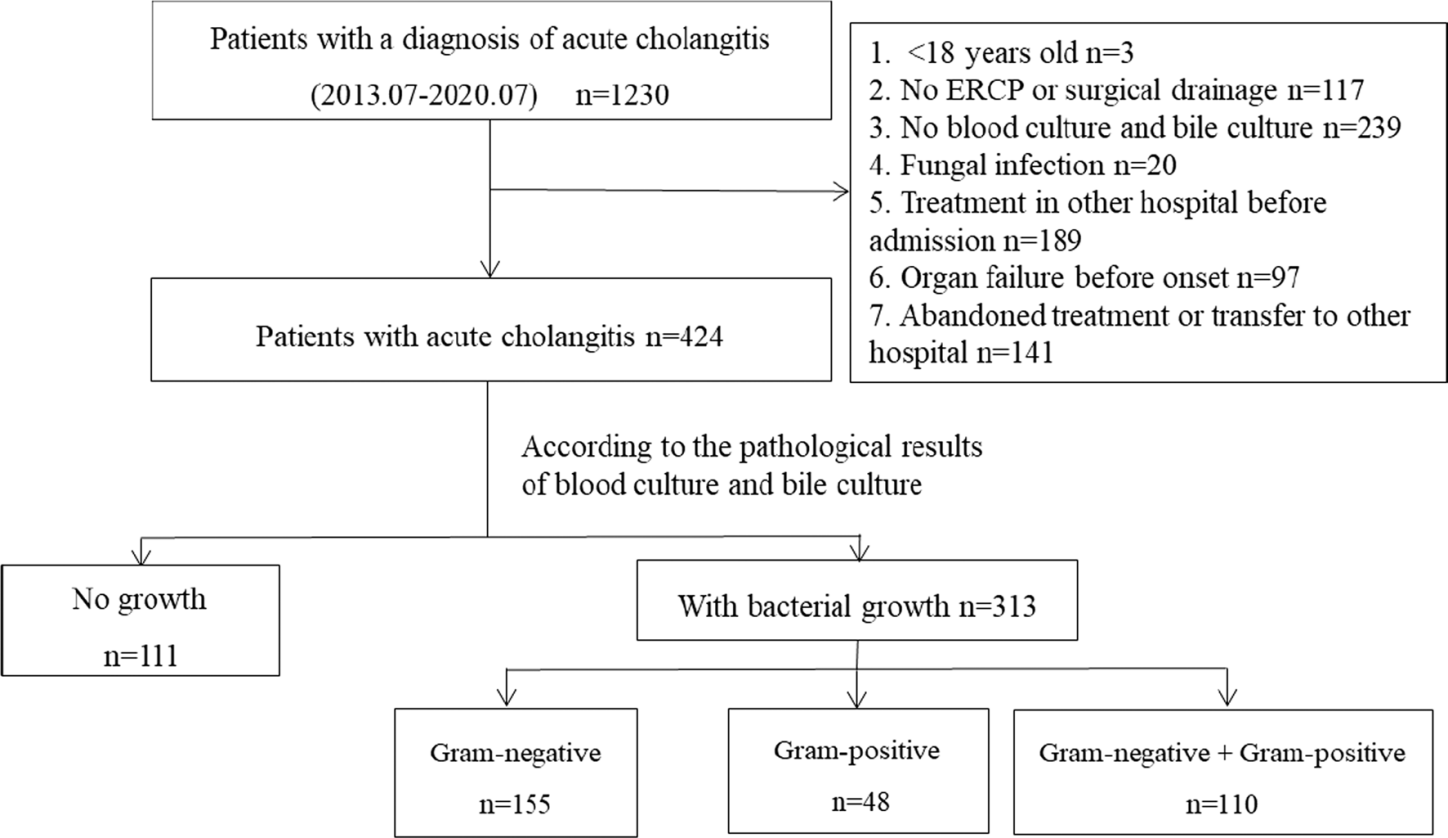
Patient selection flowchart

From July 2013 to July 2020, a total of 1230 patients were diagnosed with acute cholangitis in our hospital. After applying the eligibility criteria, 424 patients were eventually selected for the study (Fig. 1). According to the culture results, no bacterial growth developed in 111 patients (26.2%) while it developed in 313 patients (73.8%). Among the patients with bacterial growth, 155 patients had only Gram-negative bacteria cultured (49.5%), 48 patients had only Gram-positive bacteria cultured (15.3%), and 110 patients had both Gram-negative and Gram-positive bacteria cultured (35.1%). Comparing the baseline data of different culture results showed differences in gender, chronic renal insufficiency, cholecystectomy, biliary anastomosis, biliary tumors, recurrence, clinical symptoms of fever, and inappropriate antibiotic use. The baseline characteristics of patients with different culture results are summarized in Table 1.

**Table 1:** The baseline characteristics of acute cholangitis patients with different culture results

Variables		With bacterial growth			*P*-value
	No growth	Gram-negative	Gram-positive	Gram-negative + Gram-positive	
	(n = 111)	(n = 155)	(n = 48)	(n = 110)	
Age, year, median (range)	67( 51–75)	68 (56–77)	71 (58–80)	69 (54–77)	0.433
Gender, male, n (%)	60 (54.1)	80( 51.6)	15 (31.3)^a,b^	49 (44.5)	0.039
Co-morbidities/past medical history					
Cardiovascular disease, n (%)	32 (28.8)	40 (25.8)	14 (29.2)	41 (37.3)	0.245
Chronic pulmonary disease, n (%)	6 (5.4)	14 (9.0)	4 (8.3)	7 (6.4)	0.684
History of malignancies, n (%)	8 (7.2)	6 (3.9)	1 (2.0)	11 (0.1)^b^	0.120
Diabetes mellitus, n (%)	15 (13.5)	17 (11.0)	10 (20.8)	19 (17.3)	0.272
Chronic liver disease, n (%)	9 (8.1)	8 (5.2)	3 (6.3)	9 (8.2)	0.732
Chronic renal insufficiency	1 (0.9)	0 (0.0)	2 (4.2)b	5 (4.5)b	0.028
Neurologic disorder, n (%)	4 (3.6)	8 (5.2)	3 (6.3)	5 (4.5)	0.889
Connective tissue disease, n (%)	1 (0.9)	1 (0.6)	1 (2.0)	3 (2.7)	0.497
History of any biliary procedures					
Cholecystectomy, n (%)	17 (15.3)	36 (23.2)	14 (29.2)	34 (30.9)^a^	0.041
Biliary stent placement, n (%)	5 (4.5)	4 (2.6)	2 (4.2)	10 (9.1)^b^	0.113
Biliary anastomosis, n (%)	0 (0.0)	2 (1.3)	0 (0.0)	5 (4.5)	0.037
Etiology of cholangitis					
Bile duct stones, n (%)	109 (98.2)	148 (95.5)	47 (97.9)	100 (90.9)^a^	0.057
Tumor, n (%)	2 (1.8)	6 (3.9)	0 (0.0)	10 (9.1)^a^	0.017
Biliary stricture, n (%)	1 (0.9)	3 (1.9)	1 (2.0)	3 (2.7)	0.324
Recurrence, n (%)	13 (11.7)	47 (30.3)^a^	12 (25.0)^a^	49 (44.5)^a,b,c^	0.000
Symptoms					
Fever, n (%)	49 (44.1)	86 (55.5)	22 (45.8)	69 (62.7)^a,c^	0.029
Abdominal pain, n (%)	106 (95.5)	142 (91.6)	48 (100.0)^b^	101 (91.8)^c^	0.134
Jaundice, n (%)	81 (73.0)	110 (71.0)	31 (64.6)	86 (78.2)	0.322
ERCP, n (%)	51 (45.9)	68 (43.9)	16 (33.3)	51 (46.4)	0.451
Surgical operation, n (%)	60 (54.1)	87 (56.1)	32 (66.7)	59 (53.6)	0.451
Inappropriate initial antimicrobial therapy, n (%)	0 (0.0)	10 (6.5)^a^	10 (20.8)^a,b^	50 (45.5)^a,b,c^	< 0.001

^a,b,c^Used to represent the pairwise comparison results between the groups. ^a^there is a difference compared with the No growth group; ^b^there is a difference compared with the Gram-negative group; ^c^there is a difference compared with the Gram-positive group; (p < 0.05)

ERCP, Endoscopic Retrograde Cholangiopancreatography

### Microbiology

Among the enrolled 424 patients, 266 (62.7%) patients had blood culture records, and 304 (71.7%) patients had bile culture records. The positive rate of blood culture and bile culture was 52.6% (140/266), 83.2% (253/304), respectively. A total of 135 strains of Gram-negative bacilli (77.1%) were detected in blood cultures and 209 strains of Gram-negative bacilli (58.7%) in bile cultures. Among the Gram-negative bacilli, *Escherichia coli* accounted for the highest proportion, with 45.7% prevalence in blood culture and 27.0% in bile culture, followed by *Klebsiella pneumoniae*, *Enterobacter cloacae,* and *Pseudomonas aeruginosa*. There were 40 cases of Gram-positive bacilli in blood culture (22.9%) and 147 strains (41.3%) in bile culture. Among the Gram-positive cocci, *Enterococcus faecalis* and *Enterococcus faecium* accounted for the highest proportion, followed by *Enterococcus casseliflavus*, *Enterococcus gallinarum,* and *Streptococcus spp*. The results of all cultures are summarized in Table 2.

**Table 2:** Results of blood and bile cultures in patients with acute cholangitis

Species	Blood culture (%)	Bile culture (%)
Number of specimens	266/424 (62.7)	304/424 (71.7)
Positive culture	140/266 (52.6)	253/304 (83.2)
Single Gram-negative	100/266 (37.6)	114/304 (37.5)
Single Gram-positive	14/266 (5.3)	52/304 (17.1)
Gram-negative + Gram-positive	26/266 (9.8)	87/304 (28.6)
Gram-negative bacilli	135 (77.1)	209 (58.7)
*Escherichia coli*	80 (45.7)	96 (27.0)
*Klebsiella pneumoniae*	31 (17.7)	40 (11.2)
*Enterobacter cloacae*	10 (5.7)	24 (6.7)
*Pseudomonas aeruginosa*	9 (5.1)	24 (6.7)
*Klebsiella oxytoca*	2 (1.1)	1 (0.3)
*Other 2 Enterobacter spp.*	2 (1.1)	2 (0.6)
*Enterobacter aerogenes*	1 (0.6)	5 (1.4)
*Citrobacter spp.*	0 (0)	10 (2.8)
*Aeromonas hydrophila*	0 (0)	4 (1.1)
*Proteus vulgaris*	0 (0)	3 (0.8)
Gram-positive cocci	40 (22.9)	147 (41.3)
*Enterococcus faecalis*	11 (6.3)	55 (15.4)
*Enterococcus faecium*	11 (6.3)	37 (10.4)
*Enterococcus casseliflavus*	4 (2.3)	15 (4.2)
*Enterococcus gallinarum*	4 (2.3)	13 (3.7)
*Streptococcus spp.*	4 (2.3)	10 (2.8)
*Staphylococcus hominis*	2 (1.1)	1 (0.3)
*Enterococcus avium*	0 (0)	6 (1.7)
*Other Enterococcus spp.*	0 (0)	3 (0.8)
*Enterococcus raffinosus*	0 (0)	3 (0.8)
*Other Gram-positive cocci*	4 (2.3)	4 (1.1)

### The severity of illness of different cultures results

**Fig. 2:**
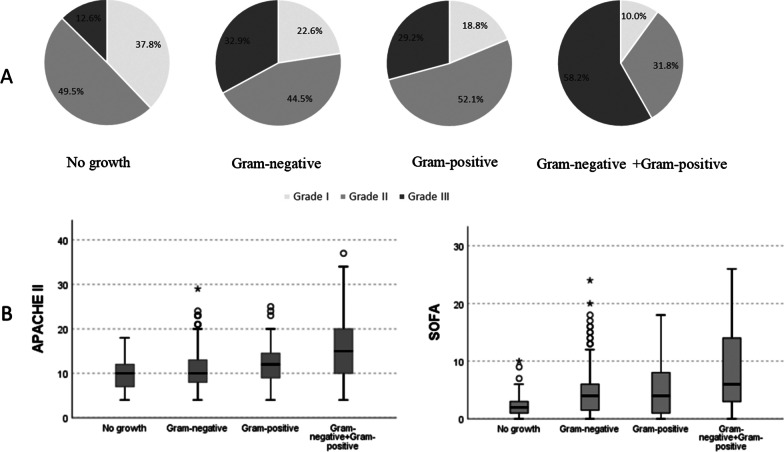
Differences in the severity of different culture results with acute cholangitis patients. **A** Using chi-square test to compare the severity of patients with different pathogen distributions (all P < 0.0001). P for trend of four groups, P < 0.0001. **B** Differences in APACHE II and SOFA scores of different pathogens. APACHE II: Acute Physiology and Chronic Health Evaluation II; SOFA: Sequential Organ Failure Assessment

The proportion of Grade III patients (58.2%) in the mixed Gram-negative and positive group was significantly higher, and Grade I patients (10%) was lower than the other three groups (P < 0.001). Additionally, the proportion of Grade III patients (12.6%) in the No-growth group was significantly the lowest, and Grade I patients (37.8%) was higher. Comparison between groups showed no difference in severity distribution between the Gram-negative group and the Gram-positive group (Fig. 2A). Moreover, the APACHE II and SOFA scores of the mixed Gram-negative and positive group were higher than the other three groups (P < 0.001), with scores of 15.00 (10.00, 20.00) and 6.00 (3.00, 14.00), respectively. The No-growth group had the lowest scores, with 10.00 (7.00, 12.00) and 2.00 (1.00, 3.00), respectively. There was no difference between the Gram-negative group and the Gram-positive group (P = 0.661, P = 1.000) (Fig. 2B). The APACHE II and SOFA scores were 10.00 (8.00, 13.00) and 4.00 (1.00, 6.00) in the Gram-negative group, 12.00 (9.00, 14.75) and 4.00 (1.00, 8.00) in the Gram-positive group, respectively.

### Organ dysfunction and the outcomes of cholangitis with different culture results

The proportion of organ dysfunction in bacterial growth patients was significantly higher than that in the No-growth patients (P < 0.05) (Table 3). The septic shock rate, neurological dysfunction, hepatic dysfunction, and respiratory dysfunction in the mixed Gram-negative and positive group was higher than the Gram-negative group. However, there was no difference between the Gram-negative group and the Gram-positive group. Hematological dysfunction and renal dysfunction of the three groups were no different (P > 0.05). In logistic regression analysis models, the bacterial growth groups had an independent risk factor for organ dysfunction (Additional file 1: Table S1). The risks of an increased septic shock, neurological dysfunction, hepatic dysfunction, hematological dysfunction, and respiratory dysfunction in the mixed Gram-negative and positive group were compared to the Gram-negative group; the odds ratio and 95% CI (confidence interval) were 2.20 (1.20 to 4.04), 3.35 (1.65 to 6.81), 2.71 (1.28 to 5.74), 1.77 (1.01 to 3.09), 3.28 (1.68 to 6.42), respectively (Table 4). There was no difference between the Gram-negative group and the Gram-positive group in organ dysfunction risk (P > 0.05). The logistic regression analysis was adjusted for the following covariates: age, gender, biliary tumor, biliary stent, cardiovascular, malignancies, diabetes, and recurrence.

**Table 3:** Differences in organ function and outcomes of cholangitis patients with different culture results

	No growth	With bacterial growth					
Variables		Total	*P*-value^c^	Gram-negative	Gram-positive	Gram-negative + Gram-positive	*P*-value^d^
	(n = 111)	(n = 313)		(n = 155)	(n = 48)	(n = 110)	
Septic shock, n (%)	3 (27.0)	70 (22.4)	< 0.001	26 (16.8)	9 (18.8)	35 (31.8)^a^	0.012
Neurological dysfunction, n (%)	4 (3.6)	55 (17.6)	< 0.001	15 (9.7)	8 (16.7)	32 (29.1)^a^	< 0.001
Hepatic dysfunction, n (%)	4 (3.6)	41 (13.1)	0.005	13 (8.4)	3 (6.3)	25 (22.7)^a,b^	0.001
Hematological dysfunction, n (%)	10 (9.0)	96 (30.7)	< 0.001	39 (25.2)	15 (31.3)	42 (38.2)	0.077
Renal dysfunction, n (%)	4 (3.6)	44 (14.1)	0.003	20 (12.9)	8 (16.7)	16 (14.5)	0.793
Respiratory dysfunction, n (%)	2 (1.8)	65 (20.8)	< 0.001	21 (13.5)	8 (16.7)	36 (32.7)^a^	0.001
Intensive care unit admission (day)	0.00 (0.00–0.00)	0.00 (0.00–2.00)	< 0.001	0.00 (0.00–1.00)	0.00 (0.00–2.00)	0.00 (0.00–3.00)^a^	0.032
Hospital stay (day)	15.00 (11.00–19.00)	17.00 (12.50–24.00)	< 0.001	16.00 (12.00–22.00)	16.50 (12.00–20.75)	21.00 (15.00–26.00)^a,b^	0.002
Death, n (%)	0 (0)	26 (8.3)	0.002	4 (2.6)	2 (4.2)	20 (18.2)^a^	< 0.001

^a,b^Used to represent the pairwise comparison results between the Gram-negative group, Gram-positive group and Gram-negative + Gram-positive group. ^a^there is a difference compared with the Gram-negative group; ^b^there is a difference compared with the Gram-positive group; (p < 0.05)

*P*-Value^c^: comparison results between the No growth group and the With bacterial growth group. *P*-Value^d^: comparison results between the Gram-negative group, Gram-positive group and Gram-negative + Gram-positive group

**Table 4:** Risk ratio of organ dysfunction in different culture results by logistic-regression model

Variables	Gram-negative	Gram-positive		Gram-negative + Gram-positive	
		Adjusted OR (95% CI)	*P*-value	Adjusted OR (95% CI)	*P*-value
Septic shock	1.0 (reference)	1.17 (0.49–2.75)	0.728	2.20 (1.20–4.04)	0.011
Neurological dysfunction	1.0 (reference)	1.71 (0.65–4.50)	0.274	3.35 (1.65–6.81)	0.001
Hepatic dysfunction	1.0 (reference)	0.75 (0.20–2.82)	0.674	2.71 (1.28–5.74)	0.009
Hematological dysfunction	1.0 (reference)	1.58 (0.75–3.32)	0.229	1.77 (1.01–3.09)	0.046
Renal dysfunction,	1.0 (reference)	1.42 (0.55–3.66)	0.472	1.12 (0.52–2.37)	0.794
Respiratory dysfunction	1.0 (reference)	1.35 (0.52–3.50)	0.542	3.28 (1.68–6.42)	0.001

Odds ratios were adjusted for age, gender, biliary tumor, biliary stent, cardiovascular, malignancies, diabetes, recurrence

CI, confidence interval; OR, odds ratios

**Fig. 3:**
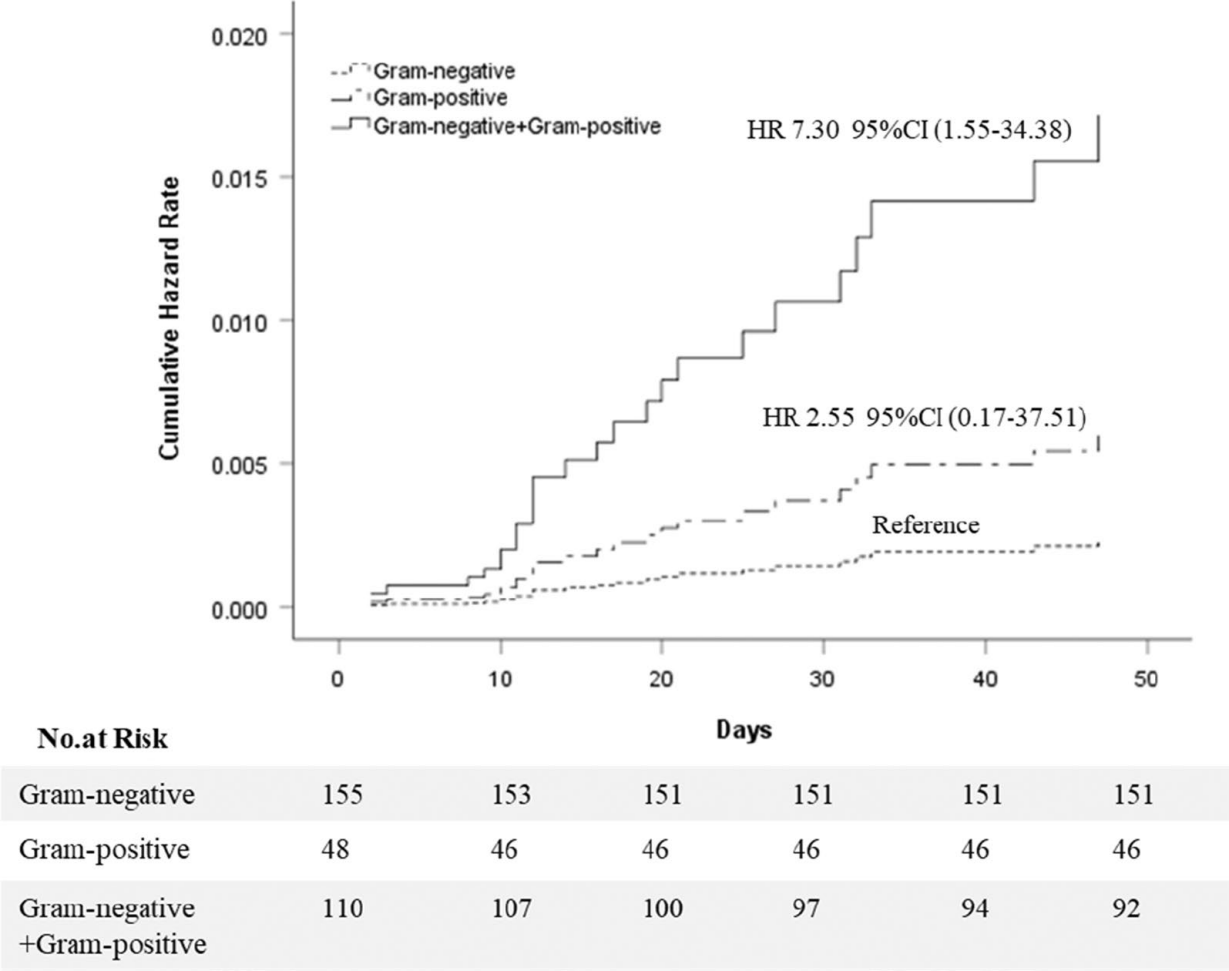
Cumulative hazard rates for death among different culture results that was the following categories: Gram-negative group, Gram-positive group, Gram-negative + Gram-positive group. 1. Cox proportional-hazards regression model were adjusted for age, gender, biliary tumor, biliary stones, biliary stent, recurrence, chronic liver disease, chronic renal insufficiency, cardiovascular, chronic-pulmonary, fever, white blood cell count, shock, neurological dysfunction, hepatic dysfunction, hematological dysfunction, renal dysfunction, respiratory dysfunction, inappropriate initial antimicrobial therapy. 2. CI: confidence interval. HR: hazard rate

The intensive care unit admission day, hospital stay, and mortality rate of the bacterial growth groups were significantly higher than the No-growth patients, with 0.00 (0.00, 2.00) and 17.00 days (12.50, 24.00), respectively, and the mortality rate was 8.3% (26/313) (P < 0.001). There was no death in the No-growth group. The intensive care unit admission day, hospital stay, and mortality rate of the mixed Gram-negative and positive group were higher than the Gram-negative group, with 0.00 (0.00, 3.00) and 21.00 days (15.00, 26.00), respectively, and the mortality rate was 18.2% (20/110) (P < 0.05). However, there were no differences between the Gram-negative group and the Gram-positive group (Table 3). The Cox proportional-hazards regression model identified different culture results as independent risk factors for death (P = 0.031). An individual rate of mixed Gram-negative and positive group showed an increased hazard ratio of 7.30 (95% CI 1.55 to 34.38, P = 0.012) compared with the Gram-negative group. The rate of hazard ratio in the Gram-positive group and the Gram-negative group was no different (P = 0.495) (Fig. 3).

## Discussion

We compared patients with acute cholangitis of different pathogenic origins regarding the severity of the disease, organ dysfunction, and the difference in prognosis. The results showed that mixed infection with Gram-negative and Gram-positive bacteria was more severe than monomicrobial negative or positive bacterial infections, more prone to organ dysfunction, and had a higher risk of death. There was no difference between the monomicrobial cultures with Gram-negative bacteria and Gram-positive bacteria.

There was no difference between Gram-negative and Gram-positive bacteria in our study regarding disease severity, organ dysfunction, and prognosis. Only a few reports in the literature compared the clinical characteristics and prognosis of acute cholangitis with Gram-negative and Gram-positive bacteria [5]. There were reports on the clinical characteristics of Gram-negative bacteria and Gram-negative bacteria in other diseases. Se Yoon Park showed that necrotizing fasciitis caused by Gram-negative pathogen had poorer outcomes than the Gram-positive counterpart [11]. Chao-Yung Yang [12] reported that the severe sepsis, septic shock, and 28-day mortality rate caused by Gram-positive and Gram-negative were the same. Ching-Yu Lee [13] also stated that Gram-negative and Gram-positive hematogenous pyogenic spondylodiscitis clinical outcomes were not different. Additionally, Zerelda Esquer Garrigo's [14] study showed no statistically significant differences in 1-year survival rates between Gram-negative and Gram-positive bacteria groups in cardiovascular implantable electronic device infections.

Gram-negative bacterial infections are the most common in acute cholangitis [15], while Gram-positive bacterial infection has gradually attracted attention recently [5, 16–19]. Moreover, many studies reported that the infection rate with Gram-positive bacteria was higher for cholangitis in liver transplantation patients than Gram-negative bacteria [20]. Lipopolysaccharides (LPS) are important outer membrane components of Gram-negative bacteria that cause disease and organ dysfunction [21]. Gram-positive pathogens can cause organ damage by releasing exotoxin and other bacterial components, such as peptidoglycan and lipoteichoic acids, which can also cause systemic inflammation [21]. Gram-negative and Gram-positive bacteria are different bacterial species, and the pathogenic toxins are different, but both types of bacteria can induce inflammation in the body and ultimately lead to organ dysfunction. So in monomicrobial infection, the effect of causing damage to the body may be the same, leading to no difference in organ dysfunction. The current guidelines for acute cholangitis address Gram-negative bacteria first, but according to the clinical manifestations and severity of the disease, Gram-positive infections also need to be considered. Monomicrobial infections pose a challenge to the choice of antibacterial drugs because many antibiotics mainly cover either gram-positive bacteria or gram-negative bacteria.

In our study, the mixed Gram-negative and Gram-positive bacteria culture rate was 35.1%, second only to Gram-negative, accounting for 49.5%, which was consistent with other studies [22]. Some studies even found that the proportion of mixed infections was higher than that of monomicrobial infections [23].

Mixed Gram-negative and Gram-positive bacterial infection in cholangitis showed the most severity, probability of causing organ dysfunction, and the worst prognosis in our study. In acute cholangitis, no existing studies compared monomicrobial bacterial infections with mixed Gram-negative and Gram-positive bacteria infections regarding organ dysfunction and prognosis. However, it has been suggested in the literature that a variety of bacteria cultured in bile may not be clinically meaningful and non-pathogenic [24]. Nevertheless, in other disease research, the same conclusions as this research were reached. M. Norizuki concluded that the combined infection of Gram-positive and Gram-negative bacteria had higher mortality than monomicrobial infection with Gram-negative or Gram-positive bacteria (57% vs. 27%) [25]. Other studies also confirmed that mixed Gram-negative and Gram-positive bacteria infection was more likely to cause septic shock and had a higher mortality rate and severity [26].

The pathogenesis of infections caused by Gram-negative and Gram-positive bacteria is not the same [21]. Mixed microbial infection of the two microorganisms could cause the pathogenic factors to work together, elevating the disease’s severity. Studies have shown that patients with mixed infection had more serious organ function damage and a higher occurrence of sepsis, which may be related to the synergistic effect of the cell wall fragments of Gram-negative and Gram-positive bacteria [26–28]. On the other hand, patients with mixed infections may have poor immunity, and once they are infected, their condition becomes more severe. Therefore, mixed infection with Gram-negative and Gram-positive bacteria should be considered in severe acute cholangitis (Grade III). This finding provides evidence to support the Tokyo Guidelines anti-infective treatment guidelines for cholangitis [6, 7], which states that if the patient is infected with severe cholangitis, antibiotic treatment should also cover Gram-positive bacteria.

The present study has some limitations as it is a single-center retrospective study with a small number of included cases and a lack of regional representation, which requires further verification by a multi-center study with larger sample size. Not all patients have completed both blood culture and bile culture, and some patients only have blood culture or bile culture. We excluded many patients who had no etiological examination, as many patients take anti-infective drugs before admission, resulting in difficulties in getting a positive culture and bias in the results. Infection with drug-resistant bacteria affects the severity of the disease, damage to organ function, and prognosis. Moreover, this study did not rule out the impact of drug-resistant bacteria on the outcome.

## Conclusion

The present study provided evidence that acute cholangitis patients with bacterial growth in bile or blood cultures were more critically ill than patients without bacterial growth, and their prognosis was worse. Mixed infection with Gram-negative and Gram-positive bacteria was more severe and prone to organ dysfunction and had higher mortality than monomicrobial infection. There was no difference between Gram-negative and Gram-positive bacterial infection in acute cholangitis regarding the severity of the disease, organ dysfunction, and prognosis.

## Supplementary Information

**Additional file 1: Table S1**. Risk ratio of organ dysfunction in different culture results by logistic-regression model.

## Data Availability

The datasets used during the current study are available from the corresponding author on reasonable request.

## Abbreviations

ERCP: Endoscopic Retrograde Cholangiopancreatography
APACHE II: Acute Physiology and Chronic Health Evaluation II
SOFA: Sequential Organ Failure Assessment
CI: Confidence interval

## References

[1] Gomi H, Takada T, Hwang TL, Akazawa K, Mori R, Endo I (2017). Updated comprehensive epidemiology, microbiology, and outcomes among patients with acute cholangitis. J Hepatobiliary Pancreat Sci.

[2] Sokal A, Sauvanet A, Fantin B, de Lastours V (2019). Acute cholangitis: diagnosis and management. J Visc Surg.

[3] Tagashira Y, Sakamoto N, Isogai T, Hikone M, Kosaka A, Chino R (2017). Impact of inadequate initial antimicrobial therapy on mortality in patients with bacteraemic cholangitis: a retrospective cohort study. Clin Microbiol Infect.

[4] Coopersmith CM, Backer DD, Deutschman CS, Ferrer R, Ishaq L, Machado FR (2018). Surviving sepsis campaign: research priorities for sepsis and septic shock. Crit Care Med.

[5] Jo IH, Kim Y-J, Chung WC, Kim J, Kim S, Lim ES (2020). Microbiology and risk factors for gram-positive Cocci bacteremia in biliary infections. Hepatobiliary Pancreat Dis Int..

[6] Gomi HA, Solomkin JSB, Takada TC, Strasberg SMD, Pitt HAE, Yoshida MF (2013). TG13 antimicrobial therapy for acute cholangitis and cholecystitis. J Hepato Biliary Pancreatic Sci..

[7] Gomi H, Solomkin JS, Schlossberg D, Okamoto K, Takada T, Strasberg SM (2017). Tokyo Guidelines 2018: antimicrobial therapy for acute cholangitis and cholecystitis. J Hepato Biliary Pancreat Sci.

[8] Kiriyama S, Kozaka K, Takada T, Strasberg SM, Pitt HA, Gabata T (2018). Tokyo Guidelines 2018: diagnostic criteria and severity grading of acute cholangitis (with videos). J Hepatobiliary Pancreat Sci.

[9] Miura F, Okamoto K, Takada T, Strasberg SM, Asbun HJ, Pitt HA (2018). Tokyo Guidelines 2018: initial management of acute biliary infection and flowchart for acute cholangitis. J Hepatobiliary Pancreat Sci.

[10] Rhodes A, Evans LE, Alhazzani W, Levy MM, Antonelli M, Ferrer R (2017). Surviving sepsis campaign: international guidelines for management of sepsis and septic shock: 2016. Intensive Care Med.

[11] Yoon PS, Nae YS, Jung LE, Tark K, Hyok JM, Ju CE (2019). Monomicrobial gram-negative necrotizing fasciitis: an uncommon but fatal syndrome. Diagn Microbiol Infect Dis.

[12] Yang CY, Lee CH, Hsieh CC, Hong MY, Chen MJ, Lee CC (2019). Differential effects of inappropriate empirical antibiotic therapy in adults with community-onset gram-positive and gram-negative aerobe bacteremia. J Infect Chemother.

[13] Lee CY, Wu MH, Cheng CC, Huang TJ, Huang TY, Lee CY (2016). Comparison of gram-negative and gram-positive hematogenous pyogenic spondylodiscitis: clinical characteristics and outcomes of treatment. BMC Infect Dis.

[14] Garrigos ZE, George MP, Vijayvargiya P, Tan EM, Farid S, Abu Saleh OM, Friedman PA (2019). Clinical presentation, management, and outcomes of cardiovascular implantable electronic device infections due to gram-negative versus gram-positive bacteria. Mayo Clin Proc.

[15] Wu ZY, Wu XS, Yao WY, Wang XF, Quan ZW, Gong W (2021). Pathogens' distribution and changes of antimicrobial resistance in the bile of acute biliary tract infection patients. Zhonghua wai ke za zhi [Chin J Surg].

[16] Doi A, Morimoto T, Iwata K (2018). Shorter duration of antibiotic treatment for acute bacteraemic cholangitis with successful biliary drainage: a retrospective cohort study. Clin Microbiol Infect..

[17] Wang D, Zhao J, He T, Wang QC, Jiang XY, Yuan Y (2020). Visualized analysis of literature on sepsis caused by Gram positive bacteria in SinoMed. Zhonghua wei zhong bing ji jiu yi xue.

[18] Reiter FP, Obermeier W, Jung J, Denk G, Mahajan UM, De Toni EN (2020). Prevalence, resistance rates, and risk factors of pathogens in routine bile cultures obtained during endoscopic retrograde cholangiography. Dig Dis.

[19] Rupp C, Bode K, Weiss KH, Rudolph G, Bergemann J, Kloeters-Plachky P (2016). Microbiological assessment of bile and corresponding antibiotic treatment: a strobe-compliant observational study of 1401 endoscopic retrograde cholangiographies. Medicine..

[20] Kabar I, Hüsing A, Cicinnati VR, Heitschmidt L, Beckebaum S, Thölking G (2015). Analysis of bile colonization and intestinal flora may improve management in liver transplant recipients undergoing ERCP. Ann Transplant.

[21] Dickson K, Lehmann C (2019). Inflammatory response to different toxins in experimental sepsis models. Int J Mol Sci.

[22] Akhtar F, Siddique MZ, Raza A, Mehmood S, Yusuf MA, Sultan F (2020). Microbiology and clinical characteristics of acute cholangitis with their impact on mortality; a retrospective cross sectional study. J Pak Med Assoc.

[23] Kruis T, Güse-Jaschuck S, Siegmund B, Adam T, Epple HJ (2020). Use of microbiological and patient data for choice of empirical antibiotic therapy in acute cholangitis. BMC Gastroenterol.

[24] Park JW, Lee JK, Lee KT, Lee KH, Sung YK, Kang CI (2014). How to interpret the bile culture results of patients with biliary tract infections. Clin Res Hepatol Gastroenterol..

[25] Norizuki M, Yamamoto S, Hosokawa N (2010). Clinical significance of polymicrobial bacteremial as determined by the pattern of gram stain. Int J Infect Dis.

[26] Dugo L, Collin M, Cuzzocrea S, Thiemermann C (2004). 15d-prostaglandin J2 reduces multiple organ failure caused by wall-fragment of Gram-positive and Gram-negative bacteria. Eur J Pharmacol.

[27] Peters BM, Jabra-Rizk MA, O'May GA, Costerton JW, Shirtliff ME (2012). Polymicrobial interactions: impact on pathogenesis and human disease. Clin Microbiol Rev.

[28] Wray GM, Foster SJ, Hinds CJ, Thiemermann C (2001). A cell wall component from pathogenic and non-pathogenic gram-positive bacteria (peptidoglycan) synergises with endotoxin to cause the release of tumour necrosis factor-alpha, nitric oxide production, shock, and multiple organ injury/dysfunction in the rat. Shock.

