# Supplementary material for: Clinical characteristics of Gram-negative and Gram-positive bacterial infection in acute cholangitis: a retrospective observational study
Source: BMC Infect Dis. 2022 Mar 20;22:269. doi: 10.1186/s12879-021-06964-1 (PMC8935737; doi:10.1186/s12879-021-06964-1)
Supplement: Supplementary file 1 — Additional file 1: Table S1. Risk ratio of organ dysfunction in different culture results by logistic-regression model. [file 12879_2021_6964_MOESM1_ESM.docx]

Table S1 Risk ratio of organ dysfunction in different culture results by logistic-regression model

|  |  | With bacterial growth | | |
| --- | --- | --- | --- | --- |
| Variables | No growth | Adjusted OR(95%CI) | *P*-Value | |
| Septic shock | 1.0(reference) | 9.03(2.75-29.69) | | <0.001 |
| Neurological dysfunction | 1.0(reference) | 5.00(1.72-14.57) | | 0.003 |
| Hepatic dysfunction | 1.0(reference) | 3.51(1.20-10.30) | | 0.022 |
| Hematological dysfunction | 1.0(reference) | 4.01(2.01-8.36) | | 0.000 |
| Renal dysfunction, | 1.0(reference) | 4.77(1.63-14.01) | | 0.004 |
| Respiratory dysfunction | 1.0(reference) | 17.17(3.72-79.24) | | <0.001 |

1.Odds ratios were adjusted for age, gender, biliary tumor, biliary stent, cardiovascular, malignancies, diabetes, recurrence.

2. CI: confidence interval. OR: odds ratios.
